# Vancouver B and C periprosthetic fractures around the cemented Exeter Stem: sex is associate with fracture pattern

**DOI:** 10.1007/s00402-021-04113-6

**Published:** 2021-08-14

**Authors:** M. F. R. Powell-Bowns, E. Oag, D. Martin, N. D. Clement, C. E. H. Scott

**Affiliations:** 1 Edinburgh Orthopaedics, Royal Infirmary of Edinburgh, Little France Cres, Edinburgh, EH164SA UK; 2 University of Edinburgh, Edinburgh, UK

**Keywords:** Total hip arthroplasty, Periprosthetic femoral fracture, Vancouver classification, Osteoporosis

## Abstract

**Introduction:**

The aim of this study was to identify factors associated with the level of periprosthetic fracture involving a cemented polished tapered stem: Vancouver B or Vancouver C.

**Methods:**

A retrospective cohort study of 181 unilateral periprosthetic fractures involving Exeter stems was assessed by three observers (mean age 78.5, range 39–103; mean BMI 27.1, 17–39; 97 (54%) male). Patient demographics, deprivation scores, BMI and time since primary prosthesis were recorded. Femoral diameter, femoral cortical thickness, Dorr classification and distal cement mantle length were measured from calibrated radiographs. Interobserver reliability was calculated using intraclass correlation coefficients (ICCs). Univariate and multivariate analysis was performed to identify associations with Vancouver B or C fractures.

**Results:**

160/181 (88%) Vancouver B and 21/181 (12%) Vancouver C-level fractures occurred at a mean of 5.9 ± 5.4 years (0.2–26.5) following primary surgery. Radiographic measurements demonstrated excellent agreement (ICC > 0.8, *p* < 0.001). Mortality was significantly higher following Vancouver C compared to B fractures: 90 day 14/160 Vs 5/21 (*p* = 0.05); 1 year 29/160 Vs 8/21 (*p* = 0.03). Univariate analysis demonstrated that Vancouver C fractures were associated with female sex, bisphosphonate use, cortical bone thickness, and distal cement mantle length (*p* < 0.05). On multivariate analysis, only female sex was an independent predictor of Vancouver C-level fractures (*R*^2 ^=0.354, *p* = 0.005).

**Conclusion:**

Most PFFs involving the Exeter stem design are Vancouver B-type fractures and appear to be independent of osteoporosis. In contrast, Vancouver C periprosthetic fractures display typical fragility fracture characteristics and are associated with female sex, thinner femoral cortices, longer distal cement mantles and high mortality.

## Introduction

The incidence of periprosthetic femoral fractures (PFFs) has been increasing over the last two decades [1]. This rise is thought to be multifactorial and includes widening indications for primary total hip arthroplasty (THA), the active and aging THA population and an increase in the proportion of the population with a THA [1]. The revision burden for this mode of failure is also increasing: 4% of revision THAs in 2014 compared to 12% in 2017 in the Scottish Arthroplasty Project [2]. The number of PFFs treated with fixation is less clear. Evidence supports that femoral stem design influences PFF risk [3–5]. Review of the joint registry data from the UK, for the most utilised cemented implants, indicates the Exeter V40 has the highest revision risk ratio of the implants as a result of fracture. However, this joint registry data fails to quantify fracture pattern or comment on patients treated with fixation [3]. Further work from the Swedish registry has compared the “composite beam” (Lubinus SP11) and to the “taper slip” (Exeter stem). This study found the Exeter stem had a greater risk of Vancouver B fractures when compared to the Lubinus SP11, yet there was no difference between the two stems for Vancouver C fractures [4]. The current evidence indicates that cementless stems have the highest rate of early revision for periprosthetic femoral fracture, and again the design of the implant (collarless cementless implant) influences the risk of revision [5].

The Exeter (Stryker, Mahwar, New Jersey, USA) femoral stem is a polished, tapered, collarless (PTC) cemented femoral stem made from stainless steel. It is the most commonly implanted cemented femoral stem in the UK constituting 69% of 467,510 cemented stems in the National Joint Registry [6]. Though it is has excellent overall survival in registry data globally [6, 7], it has been associated with an increased risk of PFF compared to anatomic cemented stems which follow different design principles [3, 4].

Polished tapered stems are designed to transmit torsional and axial load through the bone–cement interface, without creating stress shielding or excessive micro-motion [8, 9]. The philosophy behind the polished taper stem is that of loaded taper fixation [8, 9]. Tapering of the stem in two planes causes it to wedge into the cement mantle as creep occurs making it progressively more stable on axial loading, and the polished surface allows stepwise subsidence with minimal debris formation [8, 9]. Despite its success in terms of longevity [10], there is evidence of an increased risk of late PFF fracture in this design [3, 4]. This risk is thought to be further influenced by host bone [11] potentially giving cause for concern in using this stem design to treat osteoporotic hip fractures as part of THA or hemiarthroplasty constructs [11–13].

Periprosthetic femoral fractures are classified using the Vancouver system whereby the femur is first divided into zones from proximal to distal: A (at the trochanters), B (at the femoral stem) and C (distal to the stem) [14].To guide surgical management, Vancouver B fractures are further subdivided according to stem fixation and associated bone loss [14]: B1 stem well fixed; B2 stem loose; and B3 stem loose with bone loss. Though radiographic determination of stem loosening is sometimes subjective [15, 16] making the distinction between B1 and B2 fractures difficult, fracture level (B or C) is objective. While Vancouver B-level fractures often require revision arthroplasty [16, 17], C-level fractures can typically be managed with fixation.

The primary aim of this retrospective cohort study was to determine associations with periprosthetic fracture level (Vancouver B or C) in a polished tapered cemented stem design considering patient demographics, proximal femoral anatomy and cement mantle features.

## Methods

Ethical approval was obtained for this retrospective cohort study (Scotland (A) Research Ethics Committee 16/SS/0026). From 2008 to 2016, 190 consecutive unilateral Vancouver B- or C-type periprosthetic femoral fractures around cemented Exeter stems were treated at our institution and were identified from a prospectively collected trauma database. Patients were excluded if no preoperative radiograph was available for review (*n* = 9), giving a study population of 181 PFFs in 181 patients.

Medical notes including operation notes were examined and the following data recorded: demographic data, deprivation score [18], BMI, date of primary prosthesis, date of injury, mechanism of injury, and prosthesis head size (for radiographic calibration). Ninety-day and one-year mortality was calculated.

**Fig. 1:**
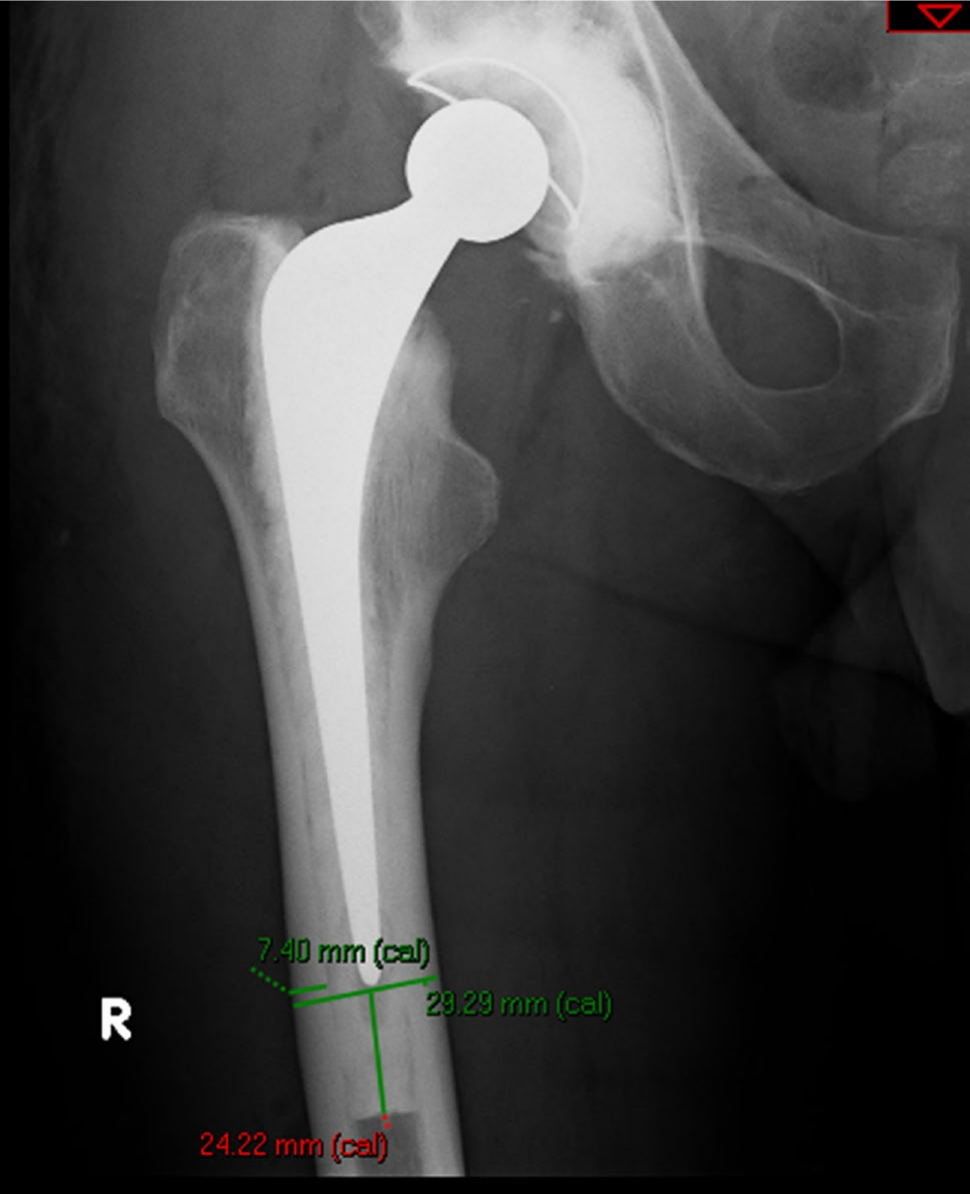
Radiographic measurements of cortical thickness, femoral diameter and length of distal cement mantle

Radiographic review was performed by three authors (MPB, EO and CEHS) who had no clinical contact with the patients. Radiographs were calibrated using femoral head size. The following were measured and recorded: Vancouver classification [14], fracture level (around the stem, at the tip, remote from the tip), fracture pattern (transverse, oblique, spiral, comminution), bone–cement interface integrity, cement restrictor type, femoral diameter (FD) and cortical thickness at the tip of the stem and distal cement mantle (DCM) length (Fig. 1). The Dorr classification [19] was calculated using the femoral (FD) and intramedullary canal (IC) diameters measured 100 mm below the tip of the lesser trochanter on an anteroposterior radiograph. Using the calculation *X* = (FD − IC)/FD, the Dorr classification was defined as: Dorr A *X* < 0.5; Dorr B *X* = 0.5–0.75; and Dorr C *X* > 0.75.

**Fig. 2:**
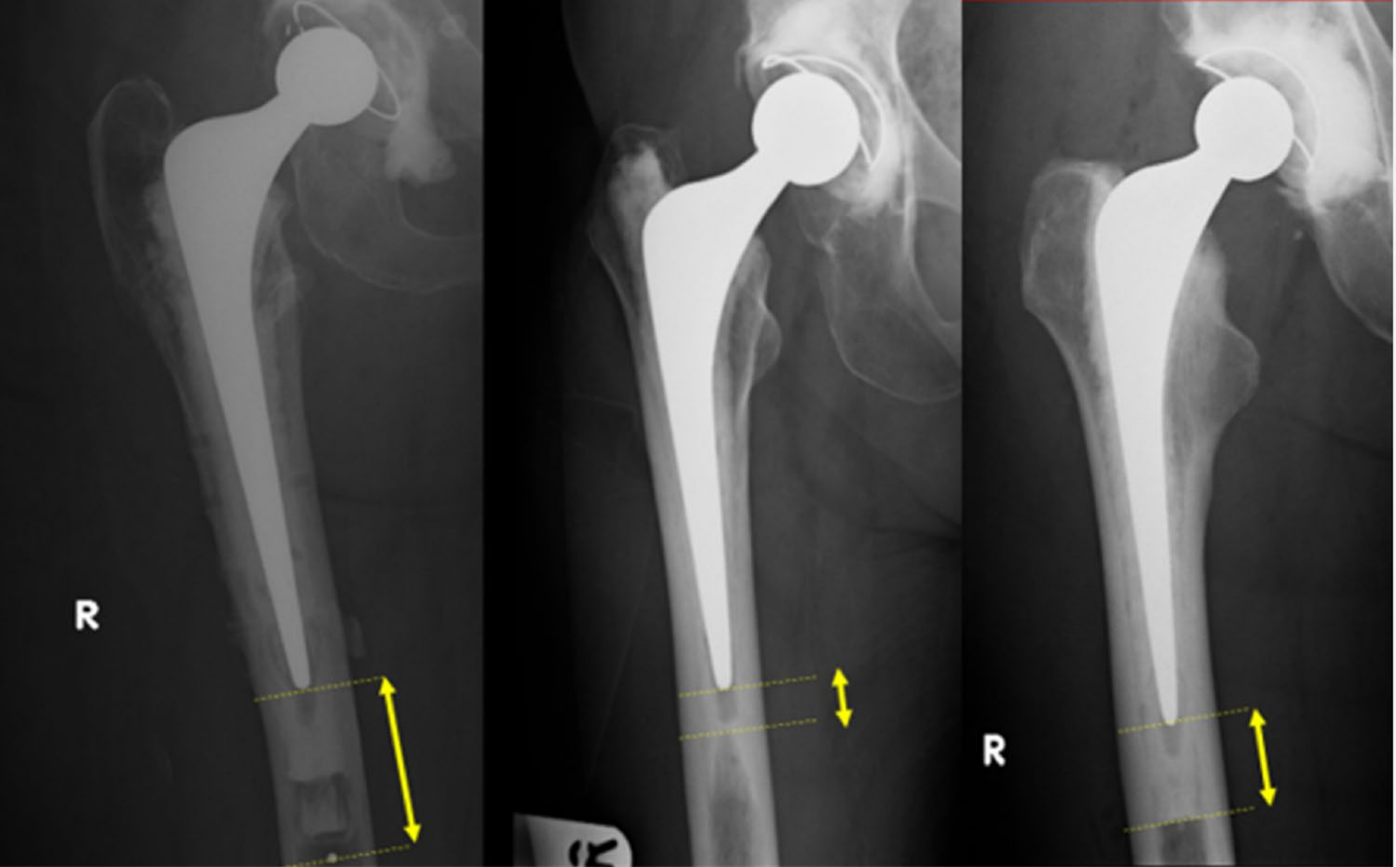
The different cement restrictors encountered in the study. From left to right: Hardinge, Exeter, Biostop. Yellow lines demonstrate how the distal cement mantle length was measured for the different cement restrictor types. For the Hardinge, the absolute distal cement mantle length was corrected to reflect folding up of the polyethylene device

Three types of cement restrictor were used: the Hardinge (DePuy, UK); the Exeter (Stryker, UK); and the Biostop (DePuy, UK). The length of the distal cement mantle was calculated differently for different cement restrictor geometry (Fig. 2). For the Biostop and Exeter restrictors, the DCM was measured from the top of the restrictor to the tip of the implant. The Hardinge cement restrictor is made of polyethylene and is designed to fold up within the intramedullary canal. This folding occurs to a greater extent in narrower intramedullary canals reducing cement mantle length relative to the marker at its distal most point (Fig. 2). To compensate for the distance between the cement mantle and the marker itself, 8 mm was subtracted from the distance measured from marker to stem tip. Where the intramedullary canal diameter was < 14 mm causing the Hardinge to fold up entirely, 20 mm (the length of the folded-up Hardinge restrictor) was subtracted from the stem tip to marker distance. Analysis was performed with both absolute and corrected DCM lengths.

### Statistical analysis

Data were analysed using SPSS version 21.0. A single-measure (two way mixed) intraclass correlation coefficient was used to quantify interobserver reliability for continuous variables (values > 0.75 = satisfactory reliability). Interobserver agreement for the Vancouver classification was calculated using the Kappa statistic. Univariate analysis was performed using parametric (Student’s *t* test: paired and unpaired) and nonparametric (Mann–Whitney *U* test) tests as appropriate to assess differences in continuous variables between Vancouver B and C fractures. Nominal categorical variables were assessed using Chi-squared or Fisher’s exact test. Pearson’s correlation was used to assess the relationship between linear variables. Variables found to be associated with Vancouver C fractures at the 10% level or less were entered stepwise into a multivariate binary logistic regression analysis using an enter methodology to identify independent predictors of distal C-type fractures. A *p* value of < 0.05 was considered statistically significant.

## Results

The study cohort consisted of 181 patients with 181 fractures around Exeter CPT stems. Characteristics of the entire cohort are given in Table 1. Sixteen (8%) stems were bipolar hemiarthroplasties, and the remainder was part of THA constructs with cemented acetabular components (Contemporary Cup, Stryker). Fourteen (8%) had no cement restrictor in situ.

**Table 1:** Patient characteristics. Mean (SD) (range), number [%]

Variable	*n* = 181
Male gender	97 [54]
BMI	27.1 (4.9) (17–39)
Age at primary (yrs)	71.1 (10.8) (30–91)
Age at fracture (yrs)	78.5 (11.5) (39–103)
Time to fracture (yrs)	5.9 (5.4) (0.2–26.5)
Primary construct	
Bipolar hemiarthroplasty	16 [9]
THA	165 [91]
Cement restrictor	
Hardinge	68 [38]
Biostop	48 [27]
Exeter	51 [28]
None	14 [8]
Management	
ORIF	152 [84]
Revision	21 [12]
Non-operative	8 [4]
Deceased	
90-day mortality	19 [10]
1-year mortality	37 [20]

Fractures occurred at a mean of 5.9 years (SD 5.4, range 0.2–26.5) following primary implantation. The mechanism of injury was a fall from standing height in 170/181 (94%); falls from height in 3 (1.5%); sport in 3 (1.5%); twisting injuries in 2 (1%); road traffic collision in 1 (1%); and 2 (1%) fractures were atraumatic. Radiolucent lines (radiographic loosening) at the bone–cement interface were present in 24 cases (13.3%).

**Fig. 3:**
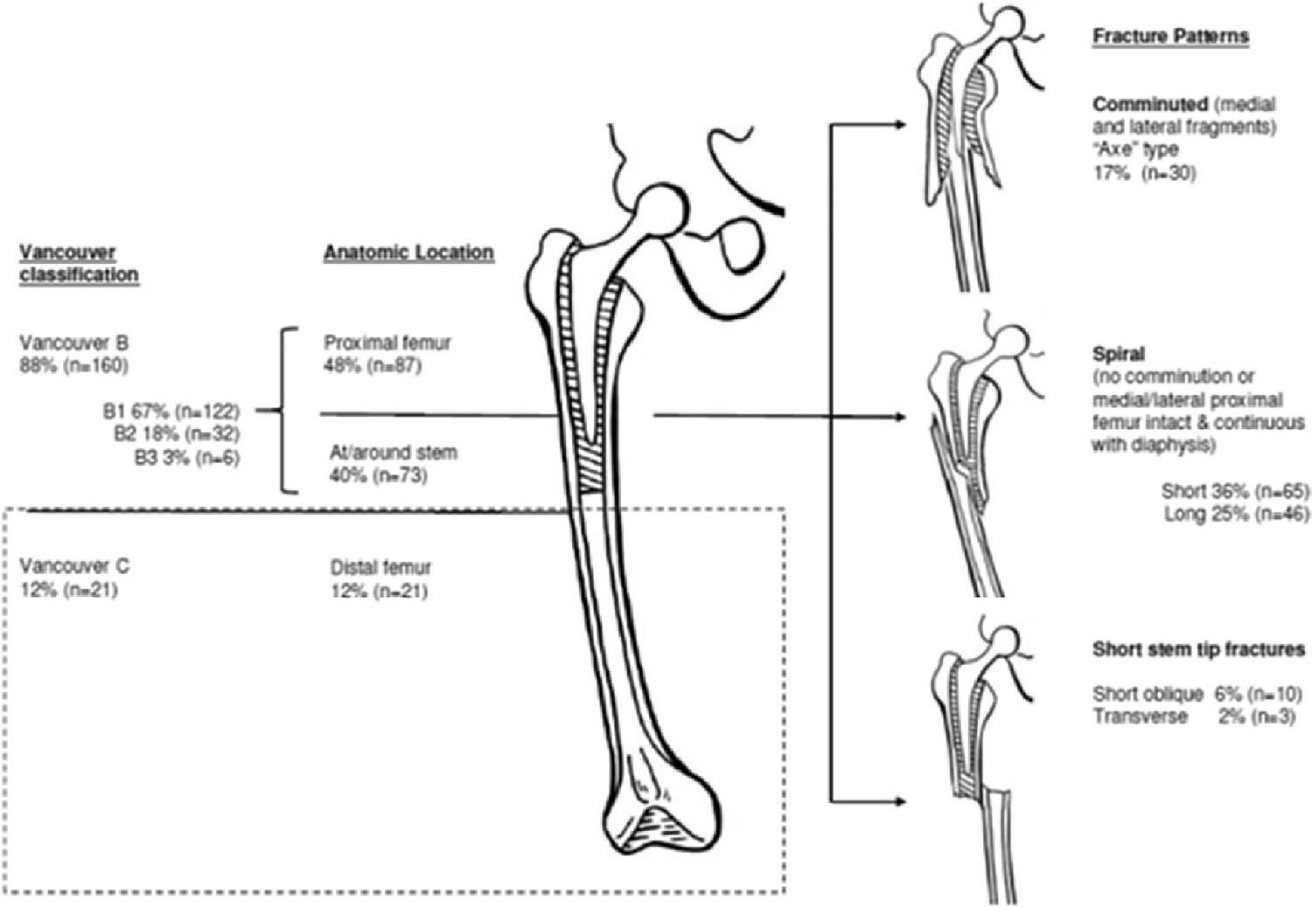
Periprosthetic fractures around the Exeter CPT stem according to the Vancouver classification and by location and pattern

The pattern, location and Vancouver classification of the fractures are displayed in Fig. 3. The Vancouver classification demonstrated strong interobserver agreement *κ* = 0.905 (*p* < 0.001). Intraclass correlations for radiographic measurements are given in Table 2. Proximal femoral cortical anatomy differed significantly by fracture level. Cortical bone thickness at the level of the stem tip was significantly less in femurs that fractured distally (Vancouver C). The distal cement mantle length was significantly longer in femurs that fractured distally (Vancouver C). When cement restrictors were present, DCM length differed significantly between cement restrictor types: longest using the Biostop (22.4 ± 12.2); shortest using the Hardinge (18.2 ± 16.1), (*p* < 0.001, ANOVA). DCM length was not significantly associated with fracture pattern (transverse/oblique/spiral). The ratios of the DCM to both the cortical bone thickness and to the femoral diameter also differed significantly between in femurs that fractured at the stem (Vancouver B) compared to those that fractured more distally (Vancouver C): longer distal cement mantles in narrower femurs with thinner cortices were associated with more distal Vancouver C-type fractures (Table 3).

**Table 2:** Intraclass correlation of radiographic measurements

Measure	Intraclass correlation	95% CI	*P* value	Cronbach’s alpha
Distal cement mantle length	0.786	0.71–0.84	< 0.001	0.800
Cortical thickness	0.878	0.84–0.91	< 0.001	0.878
Femoral diameter	0.956	0.94–0.97	< 0.001	0.978

**Table 3:** Patient characteristics by fracture level. Mean (SD), median (IQR), number [%]

Variable	Vancouver B (*n* = 160)	Vancouver C (*n* = 21)	*P* value
Female gender	65 [41]	19 [90]	< 0.001^
BMI	27.0 (4.8)	25.5 (4.0)	0.289*
Age at primary implant	70.6 (10.5)	75.4 (11.8)	0.064*
Age at fracture	78.0 (11.2)	77.4 (23.1)	0.909*
Time from index surgery to fracture	4.51 (1.3–9.3)	3.88 (1.1–7.7)	0.717**
SIMD			
1	23 [14]	3 [17]	0.534^
2	29 [18]	1 [5]	
3	22 [14]	3 [17]	
4	23 [14]	5 [25]	
5	46 [29]	6 [33]	
Comorbidities and medications			
Osteoporosis	37 [23]	8 [38]	0.140^
Steroids	17 [11]	1 [5]	0.699^^
NSAIDs	22 [14]	6 [29]	0.080^
Bisphosphonates	24 [15]	8 [38]	0.010^
Mortality			
90 day	14 [9]	5 [24]	0.050^^
1 year	29 [18]	8 [38]	0.033^
Radiographic features			
Bone–cement interface loosening	22	2	0.745^^
Femoral diameter (FD) (mm)	29.9 (4.4)	28.1 (5.0)	0.123*
Cortical thickness (mm)	6.98 (1.9)	5.96 (1.3)	0.017*
Cement restrictor			
Hardinge	61	7	0.234^
Biostop	43	5	
Exeter	46	5	
None	10	4	
Dorr classification			
A	11	0	0.436^
B	10	1	
C	139	20	
Distal cement mantle (DCM) (mm)	18.4 (12.0)	29.3 (19.1)	0.001*
DCM/cortical thickness	6.98 (1.9)	5.96 (1.3)	0.003*
DCM/FD ratio	0.62 (0.43)	0.94 (0.55)	0.007*

*Unpaired *T* test, **Mann–Whitney *U* test, ^Chi-squared, ^^Fisher’s exact

Using the Dorr classification, 20/21 (95%) distal fractures occurred in Dorr C (stovepipe) femurs. Females displayed more Dorr C-type femurs (stovepipe) 77/88 (88%) compared to males 83/99 (84%), but this was not significant (*p* = 0.088, Chi-squared). The Dorr classification did not differ significantly by deprivation level (*p* = 0.394, Chi-squared).

Univariate analysis demonstrated significant differences in fracture levels by gender, NSAID use, bisphosphonate use, cortical bone thickness, distal cement mantle length and the ratios of distal cement mantle length to both the femoral diameter and the cortical thickness (Table 3). On multivariate analysis, female gender was the only independent predictor of Vancouver C-level fractures (*R*^2^ = 0.363, B 3.1, OR 21.2 (2.4–189.1 95% CI), *p* = 0.006).

Mortality was significantly higher at both 90 days and 1 year following Vancouver C fractures compared to Vancouver B fractures (Table 3).

## Discussion

To the authors knowledge, this is the largest published single-centre series of periprosthetic femoral fractures involving the Exeter cemented polished tapered stem. The majority of fractures occurred at the level of the stem or its tip and were classified as Vancouver B (88%) fractures. Vancouver C fractures remote from the stem and its cement (12%) were associated with female sex, reduced proximal femoral cortical thickness and longer cement mantles, though female gender was the only independent predictor on multivariate analysis. Vancouver C fractures display characteristics of fragility fractures including a mortality rate of 38% at 1 year and are similar to other femoral fragility fractures such as hip fractures and periprosthetic distal femoral fractures. In this respect, they differ from more proximal Vancouver B-type fractures which have a male preponderance and frequently occur in good quality bone with thick femoral cortices. In this respect, Vancouver C fractures are likely to increase in incidence as are fragility fractures in general [1, 12, 20].

Understanding fractures around the Exeter stem is important for a number of reasons. Firstly, the Exeter is the most commonly implanted cemented femoral stem in the UK (69% of 467,510 cemented stems in the National Joint Registry [6]) and globally. Secondly, it is associated with an increased risk of PFF compared to anatomic cemented stems: twice that of the Charnley in a linked NJR study of 257,202 primary THAs [3] and ten times the risk of the Lubinus in a Swedish registry study of 65,910 primary THAs [4]. Though the absolute risk of fracture remains small (0.66% of 22,271 Exeter stems) [4], the effect of stem design is known to be more marked in patients > 80 years of age [11]. PFFs are increasing in incidence [1, 13, 16], and Vancouver B fractures are known to be associated with a 1-year mortality rate of 13–17% [21, 22]. The current study demonstrates that Vancouver C-type fractures are similar to other femoral fragility fractures and associated with a one-year mortality similar to that of hip fractures [23]

National guidelines recommend that cemented as opposed to uncemented femoral stems be used to treat hip fractures [24, 25]. Hip fracture patients have a higher risk of PFF regardless of the implant use [4]. The Exeter stem is frequently used for this indication as part of either hemiarthroplasty or THA constructs; thus, it is important to understand the implications of osteoporosis when implanting these stems. In the current study, over an 8-year period in a unit that treats > 1000 hip fractures annually, only 16 PFFs were associated with a hemiarthroplasty suggesting that use of the Exeter stem for femoral neck fracture does not generate an excessive burden of PFFs in this population.

The first element of the Vancouver classification is the proximity of the fracture to the stem. The proportion of C-level fractures (12%) reported in the current study is consistent with the literature: 10% of 1000 PFFs from the Swedish registry [4, 17] and 13% of 584 PFFs (including 237 involving the Exeter stem) from a multicentre UK-based study were C-type fractures [13]. Vancouver C-level fractures have been reported to occur more commonly with cemented composite beam-type stems (20%) than with cemented taper slip stems (12%) [13]. This may reflect ambiguity in classifying fractures around the tip of the stem—bending-type stem tip fractures are more common with composite beam compared to taper slip stems [13]. In cemented stems, technically the entirety of the cement mantle can be considered part of the stem construct. Vancouver C fractures are therefore distal to the end of the cement mantle. It appears that distal cement mantle length may influence fracture level in osteoporotic bone, possibly by moving the stress riser which occurs at the junction of stiff stem and weak bone more distally resulting in more distal fracture.

The second element of the Vancouver classification is determined by implant stability. Radiographic loosening at the bone–cement interface was present in 13% of cases at fracture in the current study. Loosening at this interface did not influence fracture level. The implant–cement interface is more difficult to apply this classification to as, by their nature, the majority of stem level fractures involve cement mantle fracture the stems are thus technically loose B2. Recent evidence suggests that such B2 fractures around cemented taper slip stems are fixable [22, 26] though revision arthroplasty is the management recommended by the Vancouver classification [14]. As this study was not assessing outcomes and it can be difficult to distinguish between B1 and B2 fractures radiographically, Vancouver B-level fractures were not further subdivided, though the level of the fracture related to the stem tip was assessed.

Though predominantly a condition of the elderly, Vancouver B-level femoral fractures affected patients of all ages including younger patients with good bone quantity as reflected by local cortical bone thickness. Though the majority of fractures in this region were spiral in nature reflecting a torsional mode of failure, there is a definite variant where the femoral stem is driven down into to the femur like a “log-splitter” causing bone and cement comminution that can be unreconstructable necessitating revision arthroplasty. This “metaphyseal split” pattern of fracture has been described previously with CCPT stem and appears unique to this stem design and typically reflects higher energy with well-fixed stems and thick femoral cortices [13, 26, 27].

Female sex has previously been recognised as a risk factor for the development of a PFF following a THA [12]. The current study has identified a preponderance of women sustaining Vancouver C-level fractures, possibly reflecting their status as fragility-type fractures. Clinicians should be aware of this, particularly in the context of an osteoporotic femur with thin cortices and “stovepipe” anatomy. There is a gradation in the stiffness of the materials that constitute the composite of a femur implanted with a cemented stem: stainless steel (230 GPa), cortical bone (21 GPa), polymethylmethacrylate (3.5 GPa) and cancellous bone (1 GPa) [28]. Stress risers occur at the junctions of construct stiffness changes. The stress riser occurring in the region of the stainless-steel femoral stem tip and its associated distal cement mantle and the native femur are exaggerated in the presence of weaker osteoporotic bone [20]. A longer distal cement mantle may graduate this change reducing this stress riser, particularly for a bending force. By altering distal cement mantle length, periprosthetic fracture biomechanics may be influenced in osteoporotic bone: employing longer distal cemented mantles in older patients with osteoporotic bone and stovepipe femurs may predispose to more distal, and therefore fixable, fractures. This may reduce the risk of Vancouver B-type fractures requiring revision arthroplasty and the high morbidity associated with this in an elderly population [22]. Leaving longer cement mantle when implanting cemented polished tapered stems in osteoporotic femurs may therefore be preferable.

Clinical studies have proposed the optimal ideal cement mantle thickness to be between 2 and 5 mm [29]. It can be difficult to achieve this in capacious Dorr-type C stovepipe femurs. There is little understanding regarding the distal cement mantle length and loosening [29, 30]. Vancouver C-type injuries were associated in the current study with stovepipe femurs and low cortical thickness, and in these patients, cement mantle length did appear to play a role. Limitations of the current study include its retrospective nature. Radiographs were not calibrated prospectively, and femoral head size was thus used as a proxy. A number of different cement restrictor designs were used, and in some historic cases, cement restrictors were absent. This created challenges in measuring distal cement mantle length and introduced assumptions into its measurement especially for the Hardinge restrictor. Cortical thickness and the Dorr classification were used as a proxy for osteoporosis. *T*-scores were not used as they were not available for many of the patients and would have limited the study numbers. It is well established that the Dorr classification and proximal femur cortical thickness can be utilised as a marker for bone quality and correlate with clinical osteoporosis [31].

## Conclusion

The majority of periprosthetic femoral fractures associated with the Exeter stem occur at the level of the stem or its tip in an elderly population. Vancouver C fractures remote from the stem were typical of fragility-type fractures and are associated with female sex, thin cortices and narrow femoral diameters. The length of the distal cement mantle and its relationship with the bony anatomy at the stem tip appeared significant in these patients, with longer cement mantles predictive of more distal, and thus fixable, periprosthetic femoral fractures. In contrast, more proximal Vancouver B fractures occurring at the level of the stem or its tip were found in patients of all ages, including younger patients with thick cortices who can display a higher energy fracture variant with comminution.
